# Reducing anti-fat bias toward the self and others: a randomized controlled trial

**DOI:** 10.1186/s40337-024-00994-1

**Published:** 2024-04-18

**Authors:** Emily B. Kramer, Evava S. Pietri, Angela D. Bryan

**Affiliations:** Department of Psychology and Neuroscience, University of Colorado Boulder, Muenzinger D244, 345 UCB, Boulder, CO 80309-0345 USA

## Abstract

Despite strong evidence linking anti-fat bias directed toward others with markers of self-directed anti-fat bias, there is a dearth of theory-based research examining the cognitive pathways underlying this relationship, and existing bias-reduction intervention efforts have thus far been conducted with exclusive focus on one domain or the other. Cognitive dissonance (CD)-based interventions have been identified as viable for reducing anti-fat bias directed toward the self and others. However, no study has yet examined whether the effects of these domain-specific interventions (e.g., anti-fat bias towards the self) extend to anti-fat bias in the non-targeted domain (e.g., anti-fat bias towards others). The present study aimed to address these gaps in the literature by comparing effects of brief CD-based interventions targeting anti-fat bias directed toward either the self or others on measures of anti-fat bias. Participants (*N* = 197, female-identifying undergraduates) were randomized to a writing activity prompting cognitive dissonance about either self-directed or other-directed anti-fat bias, or a bias-consistent control condition. Results indicated that participants in the CD intervention conditions showed significantly greater reductions in both explicit self- and other-directed anti-fat biases than those in the control condition, but results did not significantly differ across CD conditions. This supports the potential utility of interventions simultaneously targeting anti-fat bias in both domains and provides insight into the cognitive pathways underlying these related forms of bias.

**Supplementary Information:**

The online version contains supplementary material available at 10.1186/s40337-024-00994-1.

## Abstract (plain language summary)

Cognitive dissonance (CD)-based interventions have been shown to reduce anti-fat bias directed toward the self and others separately; however, no study has yet examined whether the effects of these domain-specific interventions extend to anti-fat bias in the non-targeted domain. This study aimed to address these gaps in the literature by comparing effects of brief CD-based writing task interventions targeting anti-fat bias directed toward either the self or others on a host of anti-fat bias measures. Results indicated that participants in both CD intervention conditions showed significantly greater reductions in both explicit self- and other-directed anti-fat biases than those in the control condition, which supports a potential utility of hybrid interventions simultaneously targeting anti-fat bias in both domains and provides insight into the cognitive pathways underlying these related forms of bias.

**Supplementary Information:**

The online version contains supplementary material available at 10.1186/s40337-024-00994-1.

## Introduction

Anti-fat bias and weight stigma are terms used interchangeably to refer to the social devaluation of individuals perceived to have excess bodyweight; they are pervasive and detrimental to the mental and physical health and wellbeing of their targets [44]. Negative effects include elevated rates of mood and anxiety disorders, inflammation, metabolic dysregulation, and an increased risk of death [19, 30, 41, 44]. Furthermore, a growing body of evidence suggests that experiencing weight stigma may actually lead to weight *gain* via activation of stress response systems (e.g., cortisol release), which signal to the body to store fat as protection against perceived threat [43]. Despite these harmful consequences, anti-fat bias and discrimination remain not only socially acceptable, but legally permissible and, in many cases, central to medical care practices. Given its omnipresence, it is critical to identify the primary factors underlying the development and perpetuation of anti-fat bias as a first step to eradicating it.

### Anti-fat bias and beliefs about the self

There is strong consensus among researchers that anti-fat attitudes directed toward the *self* are a consequence of internalizing the pervasive societal devaluing of the identities of individuals with stigmatized bodies [28, 34, 35]. This internalized weight bias and resulting self-stigma pose a substantial risk to the physical and mental health of the individual, as they have been shown to predict disordered eating and psychological distress [3, 16]. In addition to the elevated risks to the individual’s health and wellbeing, recent models of anti-fat bias have suggested that negative evaluations of *others* with excess bodyfat may originate from the same beliefs underlying negative evaluations of one’s *own* bodyfat (i.e., weight self-stigma or self-directed anti-fat bias; [8, 10, 12]). This duality, of internalized pressure to conform to a rigid standard and simultaneous judgment of those who do not, has been shown to exist in children as young as 8 and persist throughout adulthood, regardless of bodyweight [4, 37]. Himmelstein and Tomiyama [21] provide empirical support for this relationship, demonstrating that *self-directed* anti-fat biases influence individuals’ endorsements of negative attitudes and stereotypes directed toward *others* with excess bodyfat. Associations between anti-fat attitudes directed toward the self and others have been supported in multiple recent studies, though such studies are limited by cross-sectional designs [2, 29, 32]. Gender has also been shown to play a significant role in anti-fat bias, such that the relationship between self- and other-directed anti-fat attitudes is stronger among women than men [14, 25].

### Anti-fat bias and implicit attitudes

Anti-fat attitudes likely also stem from internalized implicit negative beliefs about bodyfat in general, encoded independently of assignment to the self or others. These implicit negative beliefs are associated with and may underlie explicit demonstrations of anti-fat bias directed towards others and the self. Research on the association between implicit versus explicit evaluative processes comes from a rich and complex theoretical tradition, but generally posits that implicit attitudes develop via cognitive processes that are distinct from the development of explicit attitudes [45].

Although research examining how implicit anti-fat attitudes relate to behavior has thus yielded mixed results, elevated implicit bias has been observed among individuals with low or no endorsement of explicit anti-fat bias on self-report measures [6, 36, 45]. This is consistent with the literature on bias in other domains (e.g., race, sexual orientation) and is believed to reflect persistent, internalized negative beliefs despite an awareness that overtly expressing negative attitudes toward members of marginalized groups is socially unacceptable. However, in contrast to implicit racial and sexuality biases, which have steadily declined since 2007, implicit anti-fat biases have remained static [7]. It is therefore critical to investigate the cognitive pathways underlying distinctly persistent implicit anti-fat attitudes to identify effective strategies to resolve them.

### Associative-propositional evaluation model

Gawronski and Bodenhausen’s [17] Associative-Propositional Evaluation (APE) Model may provide a useful framework for conceptualizing the complexities in the origins, relationships between, and perpetuation of self- and other-directed implicit and explicit anti-fat bias. The APE Model proposes two distinct, but co-occurring, internal mechanisms by which stimuli are evaluated according to pre-existing beliefs. The two processes, associative and propositional, functionally align with the development of implicit and explicit biases, respectively. The APE model distinguishes these two processes by the presence of a *truth evaluation*. More specifically, associative processes, which are posited to facilitate the development of implicit attitudes, are activated quickly, upon internalized beliefs which are not evaluated for truth or validity. By contrast, propositional processes, which are theorized to facilitate the formation of explicit attitudes, require the evaluation of the truth or validity of the belief upon which an attitude is based.

The APE model provides a particularly useful framework for conceptualizing the mechanisms underlying inconsistencies observed in explicit and implicit biases. For instance, an individual who holds an internalized belief that excess bodyweight is the direct result of laziness may react to someone they perceive as having overweight in the kickboxing class they attend several times per week with an automatic, negative associative evaluation. This evaluation is activated automatically as a result of consistent exposure to pairings of overweight with such negative qualities as laziness, thus it is not evaluated for truth or validity.

In contrast to the associative process, the propositional process is more controlled and used to develop explicit evaluations. Through this process, an individual is forced to develop and articulate an explicit evaluation of an individual with excess bodyfat. The individual is forced to reconcile their automatic negative response resulting from internalized beliefs with contradictory observations of their classmate working hard and succeeding in the high-intensity exercise sessions. Knowledge of the marginalization of people categorized as having overweight and the belief that it is wrong to discriminate against members of marginalized groups may further convince the individual to develop less negative explicit attitudes toward people perceived as having overweight and obesity. Given the validity evaluation, and subsequent rejection, of the initial core belief, it is also possible that the individual’s implicit anti-fat attitudes may shift to align with their explicit attitudes.

### Dissonance and reconciling conflict between automatic and propositional evaluations

Festinger’s [15] theory of cognitive dissonance is primarily based on the tendency for individuals to experience discomfort when their automatic evaluations of a given stimulus are inconsistent with their propositional evaluations based on relevant information or their personal values. Festinger suggested that individuals will modify the flawed beliefs underlying their automatic evaluation to be consistent with their propositional evaluation in an effort to resolve this discomfort. This principle has been incorporated into interventions in several domains by strategically prompting individuals to generate and rationalize cognitions that directly oppose an undesirable attitude or behavior.

Although this approach has not yet been applied to reduce self- and other-directed anti-fat bias in tandem, prior research suggests that it may be effective for targeting attitudes and motivation around these biases [1, 5, 9, 26, 38, 39]. Therapeutic applications of cognitive dissonance principles have predominantly been studied for reducing *self-directed* anti-fat bias and related constructs (e.g., fear of fat), which, according to a recent meta-analysis, have the most empirical support for preventing eating disorders compared with other interventional approaches [38]. Among the most effective dissonance-based interventions for reducing self-directed anti-fat bias is the Body Project, which aims to induce dissonance surrounding the marketing of diet culture, idealized thinness, and other internalized beliefs and cognitions that precede disordered eating behavior using a series of structured meetings held in a group therapy setting [1, 38]. While the results of prior studies suggest that cognitive dissonance-based therapies may hold tremendous potential for reducing explicit self-directed anti-fat bias and preventing eating disorders, their effect on anti-fat biases directed toward others has been less extensively studied.

Although the use of cognitive dissonance principles to reduce anti-fat attitudes directed toward *others* is a relatively newer application, emerging evidence suggests that it could be a promising interventional approach [11, 23, 27]. In an initial test of this approach, participants randomized to a dissonance-inducing condition showed a significantly greater reduction in explicit anti-fat attitudes, compared with a control group [9]. Despite providing evidence which favors cognitive dissonance interventions for reducing explicit anti-fat bias, the aforementioned studies did not measure implicit bias. In a similar study examining the effects of a cognitive dissonance intervention vs. a no dissonance control condition on explicit *and* implicit anti-fat attitudes, researchers observed that the dissonance group reported a reduction in explicit anti-fat attitudes following treatment, but no significant effect was observed for implicit anti-fat attitudes [5].

It is important to note that, in each of the studies described above, the cognitive dissonance intervention for reducing other-directed anti-fat bias was not comparable to that of the Body Project or other interventions more closely modeled after Festinger’s principles. More specifically, Festinger emphasized the importance of an individual *generating their own cognitions* that are incongruent with existing beliefs in order to properly induce cognitive dissonance [15]. Many of the dissonance-based interventions designed to reduce self-directed anti-fat biases, including the Body Project, incorporate this principle by facilitating discussions in which participants are prompted with questions that are strategically designed to induce dissonance by eliciting logical responses that are incongruent with existing beliefs [40]. For instance, in one Body Project exercise, participants are instructed to write a letter to a younger girl who is struggling with her body image by explaining the costs associated with pursuing the idealized thin body. In another, participants must confront the irrational expected benefits (e.g., “I’ll be happy all the time if I’m thin” and “Everyone will like me”) they associate with reaching their idealized bodyweight.

In contrast to the dissonance-based interventions aimed to reduce self-directed anti-fat bias, many of the existing dissonance-based interventions for other-directed anti-fat bias reduction do not prompt participants to generate their own dissonant cognitions. Rather, dissonance is imposed upon participants via mild researcher deception. Aside from Meaney and Rieger [27], which included an additional reflective writing task, in which participants were instructed to react to the results they had just received regarding their values and attitudes, participants *are not prompted to generate their own cognitions.* This context is critical for interpreting the results of previous studies, as they may be more reflective of demand characteristics after being informed that their responses indicated unusually high levels of anti-fat bias than any actual changes in participant’s attitudes. The inclusion of implicit bias measures can be useful for making the distinction between demand and genuine attitudinal change.

### The present study

The aims of the present research study are two-fold: to first develop an understanding of the cognitive pathways, and relevant associations, underlying the relationship between self- and other-directed anti-fat bias, and test the effects of cognitive dissonance interventions specifically targeting anti-fat bias in each domain. The relationship between self- and other-directed anti-fat bias will be explored through the lens of the associative-propositional evaluation (APE) model framework [17], which is also used to inform predictions regarding the intervention effects. Following baseline assessment of implicit and explicit attitudes about bodyweight of the self and others, participants were randomly assigned to complete a brief, online intervention aimed to induce cognitive dissonance surrounding anti-fat bias directed toward the self, cognitive dissonance surrounding anti-fat bias directed toward others, or a bias-consistent control condition where they were simply asked to confirm their existing biases.

The overarching expectation is that effects of the cognitive dissonance interventions specific to either domain (self- or other-directed anti-fat bias) will effect change to both explicit and implicit attitudes via shared evaluative pathways. A fundamental component of the APE model suggests that an existing cognitive association between two stimuli presented simultaneously will be reinforced. In this case, because participants are expected to have pre-existing, internalized associations between excess bodyfat and negative outcomes (e.g., discrimination), any information they encounter that presents individuals with excess bodyfat being treated poorly is expected to reinforce their existing beliefs. Thus, regardless of the condition-specific intervention tasks, pre-existing implicit associations between excess bodyfat and adverse experiences are expected to be strengthened among participants who read descriptions of discriminatory anti-fat policies (i.e., cognitive dissonance for other-directed anti-fat bias and control conditions). Among participants randomized to the cognitive dissonance for other-directed anti-fat bias condition, the effect of this stronger, negative association may be offset by the nature of their intervention task. Because they will be instructed to generate logical arguments that oppose discriminatory anti-fat policies, participants randomized to this condition were expected to report significant decreases in *explicit* anti-fat bias. Although either cognitive dissonance intervention could theoretically reduce bias directed towards the self and others, the APE model predicts the cognitive dissonance intervention targeting self-directed anti-fat bias should confer the greatest reduction in bias across both domains. This prediction was based on the fundamental conceptualization of self- and other-directed anti-fat biases as stemming from a common set of encoded negative associations with bodyweight. Given that the bodyweight which individuals are most frequently exposed to is likely their own, it would be expected for self-directed negative associations with bodyfat to be more frequently reinforced, and thus more strongly conditioned, than the negative associations formed in relation to the bodyfat of others. Given that strengthening these negative associations in either domain is expected to increase bias against bodyfat of both the self and others, the cognitive dissonance intervention aimed to reduce the more strongly encoded beliefs (i.e., self-directed anti-fat bias), was expected to have the most profound effect on expressed bias overall. While participants randomized to the control condition may report post-intervention decreases in explicit anti-fat attitudes, they will likely be driven exclusively by social desirability bias, and thus are expected to be weaker than in the other conditions.

**Fig. 1:**
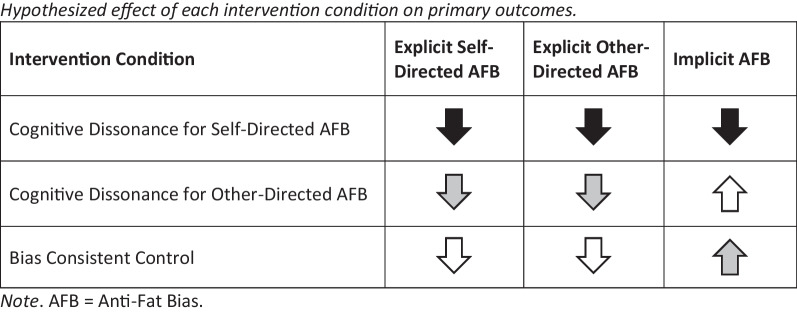
Hypothesized effect of each intervention condition on primary outcomes. *Note*. AFB = Anti-Fat Bias. Arrows reflect predicted directed of effect. Shading reflects strength of predicted effect (i.e., black = strongest effect, grey = moderate effect, white = small to nonsignificant effect)

To summarize (see Fig. 1), it is expected that participants randomized to the cognitive dissonance for self-directed anti-fat bias condition will show the greatest reduction in explicit self-directed anti-fat bias from baseline to post-intervention, followed by participants in the other-directed anti-fat bias cognitive dissonance condition, with the smallest effect observed among participants in the control condition. Participants in all three conditions will report reductions in explicit other-directed anti-fat bias from baseline to post-intervention, however, this effect will be strongest among those in the self-directed anti-fat bias condition. Implicit anti-fat bias is expected to decrease only among participants randomized to the cognitive dissonance for self-directed anti-fat bias condition.

## Method

### Design/power analysis

The present study employed a 3 condition (cognitive dissonance for self-directed anti-fat bias vs. cognitive dissonance for other-directed anti-fat bias vs. bias-consistent control) × 2 time (baseline vs. post-intervention) mixed design ANOVA where the effect of interest is the within-between interaction. Power was estimated using G*Power, which indicated that with an alpha of 0.05 and an estimated correlation of 0.50 between repeated measures, 177 participants would give a power of 0.95 to detect a small-to-moderate effect (*f* = 0.15). Allowing for an attrition rate of about 10%, which was expected based on comparable prior samples, 200 was the target for recruitment.

### Participants

The study enrolled 200 female-identifying undergraduate students through the University of Colorado Boulder subject pool, which is facilitated via the web-based SONA system. They received course credit for their participation.

Participants ranged in age from 18 to 24 (*M* = 18.58, *SD* = 0.93) and predominantly identified as white (*n* = 178, 89%). The mean BMI of the sample (*M* = 22.74, *SD* = 4.44) falls within the range categorized as “normal weight.” The majority of participants (*n* = 140, 70%) reported body sizes falling within the “normal weight” BMI classification, followed by overweight (*n* = 29, 14.5%), underweight (*n* = 20, 10%), and obese (*n* = 11, 5.5%). At baseline, most of the sample endorsed wanting to lose weight (*n* = 140, 70%) and nearly half (*n* = 98, 49.2%) reported actively pursuing weight loss.

### Procedure

Eligible individuals who expressed interest in the study registered for a designated study session via the SONA system. Participation in the study was completed remotely during a single Zoom session, which was proctored by a trained member of the research team and typically lasted a duration of 30–45 min. Participants who provided informed consent via Qualtrics survey immediately began the baseline assessment. Full descriptions of study measures are provided below, but the baseline assessment measured demographics and individual difference measures including height and weight, behavior (eating/diet and exercise behaviors), and implicit and explicit attitudes about bodyweight specific to the self (e.g., fear of fat, weight bias internalization) and others (e.g., anti-fat attitudes).

Upon completion of the baseline assessment, participants were randomized to one of the three intervention conditions (described below) and given the relevant materials and instructions to complete the intervention. Participants were allotted as much time as they needed to complete the intervention writing activity, but they were able to proceed with the study only after spending a minimum of 15 min on the activity. Following completion of the intervention session, participants immediately completed a post-intervention assessment, comprised of the same measures of implicit and explicit attitudes that were completed at baseline. Completion of the post-intervention assessment concluded study participation, and participants were provided with debriefing documents, which detailed the purpose of the study, and instructed them to return to the Zoom, where they were thanked and provided with an opportunity to ask questions.

### Interventions

In each of the three conditions, participants were asked to respond to 5 writing prompts (see Additional file 1: Appendix A). To maintain consistency with Festinger’s principles of cognitive dissonance, the dissonance-inducing exercises that comprised each intervention were modeled after components of the Body Project and other interventions which prompt participants to generate their own belief-challenging cognitions.

Participants randomized to the cognitive dissonance for self-directed anti-fat bias condition (CDself) were presented with 5 scenarios attributed to pre-teenage girls (ages 9–11) who are seeking advice on issues related to their bodyweight or body image, often including common social dilemmas (e.g., navigating peer pressure to restrict food at school, responding to incessant body comments from family members). Participants were provided with the following instructions: “Below are five submissions written by pre-teenage girls (ages 9–11) who are seeking advice about personal issues related to their bodyweight or body image. Please read each submission and provide an honest and thorough response in the corresponding text box located below. You are encouraged to draw on your own experiences, knowledge, and intuition to help craft your responses, and no outside information should be necessary.” The arguments these participants generated (e.g., advice for confronting the harmful pressures of diet culture) were expected to facilitate cognitive dissonance, as they contradict the beliefs on which their own self-directed anti-fat attitudes are based. This dissonance should have theoretically created enough internal discomfort that participants modified their previous attitudes and beliefs to align with the arguments they presented.

The second intervention condition was structurally similar to the first, but it was specifically designed to induce cognitive dissonance regarding other-directed anti-fat bias (CDother; [5, 9]). Participants were provided with the following instructions: “Below are descriptions of five legal policies. Please read each description and provide an honest and thorough response arguing ***against*** the policy in the corresponding text box located below, **regardless of your actual beliefs**. You are encouraged to draw on your own experiences, knowledge, and intuition to help craft your responses, and no outside information should be necessary.” The scenarios each described policies that are legal but specifically discriminate against individuals with overweight or obesity (e.g., social media algorithms that favor images of thin bodies, companies with size-based hiring practices that are not relevant to job duties). This was expected to generate cognitive dissonance by prompting participants to form logical arguments that directly undermine the pretenses of many common stereotypes upon which anti-fat attitudes about others are based. The dissonance should theoretically create internal discomfort among participants who hold these existing, biased beliefs, which they will reconcile by shifting to align with their logical arguments.

In the bias-consistent control condition (Control), participants were presented with the same five scenarios as the CDother anti-fat bias intervention. The instructions provided to participants in this condition were also identical, *except* participants in this condition were instructed to argue *in favor* of each policy, rather than against. This allowed for a direct, attention-matched comparison of the effects of the writing task focus among participants who were presented with the same information, differing only by argument stance. Unlike the other two conditions, the responses generated by participants in this condition were not expected to prompt cognitive dissonance because the arguments should be consistent with their pre-existing, biased attitudes and beliefs.

### Measures

*Demographics* Demographic information, including age, gender, race, BMI, socioeconomic status, and sexual orientation was collected as part of the baseline assessment.

#### Explicit other-directed anti-fat bias

*Anti-fat attitudes test (AFAT)* The AFAT is a psychometrically sound measure of *explicit other-directed anti-fat attitudes* designed for a general adult population. It is comprised of 47-items aimed to measure anti-fat attitudes in three specific domains: social/character disparagement, physical/romantic unattractiveness, and weight control/blame [22, 25]. An example item from this scale is, “I’d lose respect for a friend who started getting fat.” Each item is rated on a 5-point Likert-type response scale ranging from 1 “strongly disagree” to 5 “strongly agree,” with higher scores reflecting more negative attitudes toward fat people. Item responses were averaged to calculate a composite score of anti-fat attitudes, with higher scores reflecting greater *anti*-fat attitudes directed toward others. Internal reliability, as evaluated using Cronbach’s alpha, was strong (α = 0.9). Additionally, scores for each of the three domain-specific subscales were calculated by averaging the corresponding items.

#### Explicit self-directed anti-fat bias

*Modified Weight Bias Internalization Scale (WBIS-M)* The WBIS-M is a modified version of the Weight Bias Internalization Scale, which measures the extent to which respondents experience *self-directed anti-fat attitudes* and base self-evaluations on their weight [31]. In contrast to the original WBIS measure, which was developed specifically for use among individuals with overweight and obesity, the WBIS-M has been modified to apply to individuals across a diverse range of body sizes [13]. The scale has been shown to predict disordered eating behaviors, body image disturbances, and related psychopathologies over and above the effects of BMI. The original WBIS-M contains 11 items, however, there is strong consensus within prior literature that the first item should be dropped due to low reliability [20, 24]. Thus, the WBIS-M used in the present study contains the remaining 10 items, which are rated on a 7-point Likert-type scale ranging from 1 “strongly disagree” to 7 “strongly agree.” An example item is “My weight is a major way that I judge my value as a person.” Item responses were averaged to calculate a composite score of weight bias internalization, with higher scores reflecting greater internalized weight bias (α = 0.94).

*Goldfarb Fear of Fat Scale (GFFS)* The GFFS was initially developed for diagnostic purposes to evaluate fear of fat or weight gain among patients with clinically significant bulimia, but it has recently been psychometrically validated for use in non-clinical, female samples [18, 33]. This was used in addition with the WBIS-M to measure *self-directed explicit anti-fat bias*. In contrast to weight bias internalization, which measures an individual’s evaluation of their weight status at present, the GFFS measures preoccupation with future weight gain. Fear of fat has been identified as a fundamental motivator of disordered eating behaviors driven by body-image dissatisfaction, particularly among women, and it was used in this study as an additional measure of self-directed anti-fat attitudes [8]. The scale contains 10 items, such as “Becoming fat would be the worst thing that could happen to me,” which were rated on a 4-point Likert-type response scale ranging from 1 “very untrue” to 4 “very true,” with higher scores reflecting greater fear of fat (α = 0.85).

#### Implicit anti-fat bias

*Weight (“Fat*–*Thin”) Implicit Association Test (IAT)* The Thin–Fat IAT is a timed, online association task that has been developed and validated for measurement of *implicit anti-fat attitudes* among the general population [42]. Participants are shown a series of silhouette images and individual words one at a time, with instructions to categorize them as quickly as possible according to body size (thin/fat) and connotation (positive/negative), respectively. The categorization system changes mid-way through the task, such that each body size category is paired with each word connotation. Differences in latency measurements for categorizing “fat” vs. “thin” images with positive words are used to quantify implicit anti-fat attitudes.

## Analyses

Baseline descriptive statistics were first calculated and compared among randomization conditions using one-way ANOVAs. Additional analyses of baseline data were conducted via bivariate correlations between measures of each form of bias—explicit self-directed anti-fat attitudes (WBISM and GFFS), explicit other-directed anti-fat attitudes (AFAT), implicit anti-fat attitudes (IAT), body-mass index (BMI) and intervention condition.

Multilevel models were next estimated to test the effects of time (pre-intervention vs. post-intervention), intervention condition (CDself vs. CDother vs. Control), and their interaction on each measure of anti-fat bias (GFFS, WBISM, AFAT, IAT). In each model, intervention condition was contrast coded, such that the effect of randomization to either cognitive dissonance intervention group (CDboth) were first compared with the control, then the effect of randomization to the Self-Directed Cognitive Dissonance (CDself) condition was compared with the Other-Directed Cognitive Dissonance (CDother). Simple effects tests were conducted in the case of significant condition X time interaction effects.

## Results

Baseline demographic characteristics by intervention condition are presented in Table 1. The percentage of participants who identified as Hispanic or Latina significantly differed by randomization condition, thus it was included as a covariate in analyses. Otherwise the three conditions were equivalent. Bivariate correlations between each of the measures of anti-fat bias and BMI are presented in Table 2. Explicit other-directed anti-fat bias (AFAT) was positively correlated with explicit self-directed anti-fat bias and implicit anti-fat bias. The two measures of explicit self-directed anti-fat bias, GFFS and WBISM, were significantly correlated with each other, but neither was significantly correlated with implicit anti-fat bias. Body-mass index (BMI) was positively correlated with both measures of explicit self-directed anti-fat bias, and negatively correlated with implicit anti-fat bias, suggesting that participants of higher body weight had more negative explicit self-directed biases but more positive implicit attitudes. BMI was not significantly related to explicit other-directed anti-fat bias.

**Table 1:** Baseline demographics, sample characteristics, and anti-fat bias by condition

	Full sample	CD for self-directed AFB	CD for other-directed AFB	Bias-consistent control	*F* or χ^2^	*p*
	*M* (*SD*) or No. (%)					
*N*	198	67	67	64		
Age	18.58 (.93)	18.76 (1.12)	18.52 (.77)	18.45(.87)	3.63	.058
Race						
AI/AN	4 (2.02%)	0 (0%)	1 (1.49%)	3 (4.69%)		
Asian	26 (13.13%)	11 (16.42%)	7 (10.45%)	8 (12.5%)		
Black or AA	2 (1.01%)	1 (1.49%)	1 (1.49%)	0 (0%)		
White	178 (89.9%)	62 (92.54%)	60 (89.55%)	54 (84.38%)		
Hispanic/Latina	28 (14.07%)	**1 (1.52%)**	13 (19.4%)	13 (20.31%)	9.13	**.003**
Sexual orientation^a^	142 (71%)	49 (73.1%)	43 (64.2%)	48 (75%)	.046	.831
BMI	22.74 (4.44)	22.68 (4.44)	22.69 (4.32)	22.85 (4.7)	.047	.829
Want to lose weight	138 (69.7%)	48 (71.6%)	43 (64.2%)	47 (73.4%)	.043	.837
Trying to lose weight	96 (48.7%)	31^b^ (47.0%)	31 (46.3%)	34 (53.1%)	.048	.488
AFAT	1.49 (.32)	1.5 (.29)	1.45 (.27)	1.54 (.38)	.398	.529
GFFS	19.44 (6.03)	19.3 (5.35)	18.92 (6.27)	19.69 (6.3)	.259	.611
WBISM	3.2 (1.5)	3.12 (1.45)	3.11 (1.49)	3.33 (1.56)	.634	.427
IAT *D*-Score^c^	0.43(0.38)	0.41 (0.41)	0.5 (0.35)	0.41 (0.4)	.043	.836

Bold values denote statistically significant differences across randomization condition at the *p* < .01 level

*CD* cognitive dissonance, *AFB* anti-fat bias, *AI/AN *American Indian/Alaska Native, *AA* African–American, *AFAT* anti-fat attitudes, *GFFS* global fear of fat scare, *WBISM *weight bias internalization, *IAT* implicit association test

^a^Sexual Orientation in terms of *n* identified as straight/heterosexual (%)

^b^Missing response for *n* = 1 participant

^c^IAT *D*-Scores were calculated such that more positive scores indicate greater implicit anti-fat bias, and more negative scores indicate greater implicit anti-thin bias

**Table 2:** Intercorrelations between baseline measures of anti-fat bias, body-mass index, and randomization condition

	AFAT	GFFS	WBISM	IAT *D*-score	BMI
AFAT	–				
GFFS	0.44***	–			
WBISM	0.20**	0.65***	–		
IAT *D*-Score^a^	0.28***	0.04	− 0.01	–	
BMI	− 0.05	0.14*	0.33***	− 0.15*	–

*AFAT* anti-fat attitudes, *GFFS* global fear of fat scare, *WBISM* weight bias internalization, *IAT* implicit association test, *BMI* body-mass index

^a^IAT *D*-scores were calculated such that positive scores indicate greater implicit anti-fat bias, and negative scores indicate greater implicit anti-thin bias

*p < .05. **p < .01. ***p < .001

### Multilevel modeling to test intervention effect on anti-fat bias

Pre- and post-intervention means of primary outcome variables by condition appear in Table 3.

**Table 3:** Measures of anti-fat bias by intervention condition and assessment timepoint

Variable	CD self-directed			CD other-directed			Control		
	*n*	Mean	*SD*	*n*	Mean	*SD*	*n*	Mean	*SD*
AFAT									
Pre	63	1.50	0.29	64	1.45	0.27	62	1.54	0.38
Post	63	1.46	0.27	64	1.38	0.24	62	1.52	0.39
GFFS									
Pre	63	19.30	5.35	64	18.92	6.27	62	19.69	6.30
Post	63	18.40	5.92	64	17.91	6.98	62	19.53	7.24
WBISM									
Pre	63	3.12	1.45	64	3.11	1.49	62	3.33	1.56
Post	63	2.90	1.43	64	2.88	1.46	62	3.20	1.59
IAT *D*-Score^a^									
Pre	63	0.41	0.41	64	0.50	0.35	62	0.41	0.40
Post	63	0.26	0.38	64	0.38	0.36	62	0.30	0.36

*CD* cognitive dissonance, *AFAT* anti-fat attitudes, *GFFS* global fear of fat scare, *WBISM* weight bias internalization, *IAT* implicit association test

^a^IAT *D*-Scores were calculated such that more positive scores indicate greater implicit anti-fat bias, and more negative scores indicate greater implicit anti-thin bias

Results of the multilevel model estimations are presented in Additional file 1: Tables S1–S4. Given the significant difference in number of participants who identified as Hispanic or Latina across condition, sensitivity analyses were conducted to examine whether results differed when controlling for self-identification as Hispanic or Latina. The results of analyses including this covariate were all consistent with those without any covariates, thus the results of the simpler analyses are reported below.

#### Self-directed anti-fat bias

For GFFS, there was a significant time by condition (control vs. either cognitive dissonance condition) interaction, such that participants randomized to either of the cognitive dissonance conditions showed a 0.45-point greater reduction in anti-fat bias from baseline to post-assessment than their counterparts in the control condition. There was no significant difference in the amount by which GFFS scores decreased among participants in the two cognitive dissonance conditions. For the other measure of self-directed anti-fat bias, weight bias internalization (WBISM), there was a significant effect of time, such that, on average, participants in all three conditions demonstrated a 0.09-point decrease in internalized weight bias from baseline to post-intervention, but there were no significant differences by condition.

#### Other-directed anti-fat bias

In the model predicting explicit other-directed anti-fat bias (AFAT), there were significant effects of time and a time by condition (control vs. either cognitive dissonance condition) interaction, such that AFAT scores decreased significantly for all participants from baseline to post-intervention, but participants randomized to either of the cognitive dissonance conditions showed significantly greater reductions in anti-fat bias, averaging a 0.02-point greater decrease, from baseline to post-assessment, than their counterparts in the control condition.

Exploratory analyses were then conducted to further examine whether the observed effect on AFAT scores is attributable to one of the specific AFAT subscales. The significant effects of time and the time by condition (control vs. either cognitive dissonance condition) interaction were replicated only in the model predicting Physical/Romantic Unattractiveness subscores. In the model predicting the Weight Control/Blame component, there was a significant effect of time, such that participants across all three conditions showed a significant decrease in anti-fat attitudes specific to weight control/blame from baseline to post-intervention. No significant effects of time, condition, or their interaction were observed for the Social/Character Disparagement subscale.

#### Implicit anti-fat bias

Finally, in the model predicting implicit anti-fat bias (IAT), there was a significant effect of time, such that, on average across all conditions, participants exhibited a 6% reduction in implicit anti-fat bias from baseline to post-intervention, with no significant difference by condition.

Exploratory analyses were then conducted to examine whether the correlation between implicit bias and each measure of explicit bias changed significantly from baseline to post-intervention. The correlation between explicit other-directed anti-fat bias (AFAT) and implicit anti-fat bias (IAT) decreased from *r* = 0.28 at baseline to *r* = 0.18, with only marginal significance at post-intervention, *F*(1, 188) = 3.87, *p* = 0.051. The correlations between IAT and each of the measures of explicit self-directed anti-fat bias (GFFS and WBISM) remained non-significant at post-intervention, *p*’s > 0.201.

## Discussion

In accordance with predictions derived from the associative-propositional evaluation (APE) model, strong correlations were observed at baseline between explicit other-directed anti-fat bias and both explicit self-directed anti-fat bias and implicit anti-fat bias. Explicit self-directed anti-fat bias was not, however, significantly correlated with implicit anti-fat bias at baseline. This suggests that further examination of the potentially divergent cognitive pathways underlying the activation of these forms of bias is needed. This nonsignificant relationship may indicate that components of implicit anti-fat bias and explicit self-directed anti-fat bias develop from distinct cognitive processes, and thus the simple silhouette images used to evaluate implicit bias in the IAT may not have caused participants to draw on their beliefs and attitudes about their own bodyweight. Additionally, the reduction in the correlation strength between explicit other-directed anti-fat bias and implicit anti-fat bias at post-intervention may reflect the variations in length of time required to change cognitions that are formed via different underlying evaluative processes.

It is worth noting though that the observed effects, or lack thereof, may also be related to psychometric issues in the present study, particularly regarding explicit self-directed anti-fat bias. Despite the inclusion of two measures of explicit self-directed anti-fat biases in this study, these existing measures actually capture only a narrow portion of what may be included in this broader construct. For example, the WBISM primarily probes the extent to which individuals evaluate themselves negatively or anticipate social ostracization *because* of their weight (e.g., “Because of my weight, I don’t understand how anyone attractive would want to date me”). Thus, higher scores on this measure of self-directed anti-fat bias indicate that participants consider their body to have excess weight *and* they feel negatively about it. The GFFS focuses more on feelings associated with hypothetical future weight gain (e.g., “Becoming fat would be the worst thing that could happen to me”). While these two scales do measure important aspects of self-directed anti-fat bias, they fail to comprehensively capture the extent of participants’ preoccupation with and valuation of maintaining a thin physique. Given that the vast majority of participants reported wanting to lose weight, despite the average BMI of the sample falling within the range categorized as “healthy,” it is important to further examine the cognitions, beliefs, and accompanying self-talk that underlie this pervasive drive for thinness. For example, it would be helpful to understand the benefits participants associate with having a thin body (e.g., social inclusion, relationship success), the sacrifices they make to pursue or maintain thinness (e.g., skipping social events where they may feel pressure to break their diet), and their affective responses to subtle body changes, such as small fluctuations in weight.

With regard to the second goal of the study, to examine the effects of cognitive dissonance interventions, there was a significant main effect of time for all four outcome measures of anti-fat bias indicating that bias was reduced after exposure to any of the three conditions. However, the strength of this effect differed by condition on some outcomes. Only participants randomized to one of the cognitive dissonance intervention conditions experienced significant reductions in explicit other-directed anti-fat bias (AFAT) and the fear of fat component of self-directed anti-fat bias (GFFS). The lack of observed differences by condition on implicit anti-fat bias (IAT) and the weight bias internalization component of self-directed anti-fat bias (WBISM) may be explained by the APE model supposition that the associative processes underlying implicit or internalized beliefs are formed rather slowly, requiring exposure to many repeated pairings of different associations to experience actual change.

The overall effects of time, such that bias improved across all three conditions, may thus be indicative of a salient sympathy that was activated by exposure to any of the writing prompts or even the baseline measures. With regard to the WBISM specifically, participants may have endorsed the items less strongly following the intervention because their experiences of weight-related social mistreatment and internal duress seemed less severe in comparison to the examples of anti-fat bias they had just read. Furthermore, the overall reduction observed in implicit bias scores could simply reflect a practice effect on the implicit association task. Although a practice effect on the implicit association task is possible, prior research has found implicit bias to remain stable following a brief intervention, despite observing a significant reduction in explicit anti-fat bias across the same assessment timepoints [5]. This suggests that the significant reduction in IAT scores in the present sample may reflect meaningful change in implicit bias following exposure to any of the interventions.

The intervention effects observed across cognitive dissonance conditions from baseline to post-assessment for explicit other-directed anti-fat bias (AFAT) and the fear of fat component of self-directed anti-fat bias (GFFS) further uphold the predictions of the APE model, given that these attitudes stem from propositional processes, which can change more quickly with new information. The non-significant differences in effects across the two cognitive dissonance conditions (one meant to be more self-focused and one meant to be more other-focused) requires further examination. The cognitive dissonance for self-directed anti-fat bias intervention content focused entirely on behaviors typically connected to bodyweight (e.g., diet, exercise); whereas the cognitive dissonance for other-directed anti-fat bias intervention content focused specifically on appearance-related discrimination. Notably, results of the exploratory analyses of the AFAT subscales aligned with this focus, suggesting an effect of the interventions as a whole on both appearance and specific behavior-related biases (i.e., Physical/Romantic Unattractiveness and Weight Control/Blame subscales), but not on the Social/Character Disparagement subscale. While it is possible that the cognitive associations underlying anti-fat biases (i.e., appearance-based attitudes) may be encoded separately from our cognitive associations with diet and exercise behaviors, the findings from this study suggest that anti-fat attitudes stemming from cognitions about behavior may actually be conflated with cognitions about appearance, but appearance-related cognitions may be more readily manipulable. Stronger manipulations meant to distinguish these two sources of bias will be important in future research. Specifically, and in accordance with the logic underlying the present study hypotheses, future studies should examine whether the addition of more concrete *appearance-specific* stimuli (e.g., description of the writer’s size or inclusion of a picture) to the self-directed anti-fat bias intervention prompts produce greater reductions in explicit anti-fat biases.

### Limitations and future directions

Given the lack of distinction in the effects of the two cognitive dissonance intervention conditions on anti-fat bias, further research is required to develop the optimal cognitive dissonance intervention for reducing anti-fat biases directed toward the self and others. This optimal intervention may well be a hybrid of the interventions used here to prompt cognitive dissonance about anti-fat bias directed toward *both* the self and others with examples that may relate to both behavior (e.g., diet, exercise) and appearance (e.g., discussion of body size classifications with no evidence of behavioral/health data). Refinement of this intervention may benefit from further research examining whether the inclusion of images of individuals linked to the intervention writing prompts cues different responses depending on the body sizes of the authors.

Furthermore, more formal qualitative analyses of the participant intervention writing task responses are ongoing but beyond the scope of the present analyses. A brief fidelity check was conducted by multiple members of the research team to assure that responses broadly appeared to align with the assigned task; however, future analyses of patterns and themes observed in the responses across conditions may aid in our understanding of the cognitive processes underlying each of the intervention effects.

Additionally, future studies must utilize measures that evaluate explicit self-directed anti-fat bias more comprehensively, which may first require a stronger consensus among researchers regarding the definition of this construct and psychometric approaches to measuring it. The development of definitions and psychometric instruments to measure this construct should aim to capture the broad scope of cognitions, beliefs and attitudes that are indicative of anti-fat bias.

Relatedly, there is a potential for measurement bias in the results drawn from self-report data, particularly given the sensitive nature of the topic, which may have prompted participants to respond as they believed they *should*, rather than with their actual beliefs. Given that social desirability was not measured in the present sample and thus cannot be quantified or otherwise accounted for, the results of the present study must be interpreted cautiously.

There are additional potential limitations to the generalizability of the findings to populations beyond the relatively homogenous sample included in the present study. In particular, there is some evidence to suggest that body size could play a role in the way individuals receive and engage with material regarding weight and stigma [37]. Thus, given that the majority of the present sample would be classified as having a BMI in the “healthy” range, the results may not generalize to individuals with more diverse body sizes. Additionally, despite the known gender differences in anti-fat attitudes and beliefs, bias reduction research efforts must be expanded beyond all-female samples. Although women are disproportionately targeted by weight stigma, anti-fat beliefs and rhetoric are pervasive on a societal level, which poses a serious threat to the mental and physical health of those who internalize the messaging. This demands similarly scaled, population-level interventions to disrupt.

Finally, the long-term effects of the present study interventions are unknown, due to the single intervention exposure and lack of follow-up assessment. Future studies should incorporate longitudinal designs and consider testing whether repeated exposure to the intervention produces a greater effect, via additional reinforcement of the cognitive uncoupling of bodyfat with implicit and explicit negative associations. To ensure engagement, particularly with multiple intervention exposures, future iterations of the intervention could be designed for use with a smartphone app or other tech format that is appealing and easily accessible to its targeted users.

### Supplementary Information

**Additional file 1.** Supplementary information presented in Additional file 1 includes the writing prompts assigned to each intervention condition (Appendix A) and results of multilevel models estimated for each of the primary analyses (Table S1–S4).

## Data Availability

The study protocol and de-identified participant data collected during the trial is available upon request. Data will be available beginning 3 months and ending 5 years following article publication. Data will be shared with researchers who provide a methodologically sound proposal. Proposals should be directed to emily.kramer-1@colorado.edu. To gain access, data requesters will need to sign a data access agreement.
