# Supplementary material for: Reducing anti-fat bias toward the self and others: a randomized controlled trial
Source: J Eat Disord. 2024 Apr 18;12:46. doi: 10.1186/s40337-024-00994-1 (PMC11025213; doi:10.1186/s40337-024-00994-1)
Supplement: Supplementary file 1 — Additional file 1. Supplementary information presented in Additional file 1 includes the writing prompts assigned to each intervention condition (Appendix A) and results of multilevel models estimated for each of the primary analyses (Table S1–S4). [file 40337_2024_994_MOESM1_ESM.docx]

**Additional file 1**

**Table S1.**

*Results of Multilevel Model Testing Effect of Time by Condition Interaction on GFFS.*

| *Predictors* | *Estimates* | *CI* | *p* |
| --- | --- | --- | --- |
| (Intercept) | 19.01 | 18.13 – 19.89 | **<0.001** |
| time | -0.38 | -0.55 – -0.21 | **<0.001** |
| control_VS_CDboth | 1.42 | -0.46 – 3.30 | 0.139 |
| CDself_VS_CDother | -0.79 | -2.93 – 1.35 | 0.467 |
| **time * control_VS_CDboth** | 0.45 | 0.08 – 0.82 | **0.017** |
| time * CDself_VS_CDother | 0.03 | -0.39 – 0.45 | 0.879 |
| **Random Effects** | | | |
| σ^2^ | 3.05 | | |
| τ_00_ _ResponseId_ | 38.08 | | |
| ICC | 0.93 | | |
| N _ResponseId_ | 198 | | |
| Observations | 396 | | |
| Marginal R^2^ / Conditional R^2^ | 0.018 / 0.927 | | |

*Note*. GFFS = Global Fear of Fat Scale. CDboth = Group comprising those randomized to the Self-Directed Cognitive Dissonance and Other-Directed Cognitive Dissonance conditions. CDself = Self-Directed Cognitive Dissonance. CDother = Other-Directed Cognitive Dissonance.

**Table S2.**

*Results of Multilevel Model Testing Effect of Time by Condition Interaction on WBISM.*

| *Predictors* | *Estimates* | *CI* | *p* |
| --- | --- | --- | --- |
| (Intercept) | 34.12 | 32.03 – 36.20 | **<0.001** |
| **time** | -1.19 | -1.55 – -0.83 | **<0.001** |
| control_VS_CDboth | 2.63 | -1.83 – 7.08 | 0.247 |
| CDself_VS_CDother | -0.32 | -5.39 – 4.74 | 0.9 |
| time * control_VS_CDboth | 0.37 | -0.41 – 1.14 | 0.352 |
| time * CDself_VS_CDother | -0.17 | -1.05 – 0.71 | 0.696 |
| **Random Effects** | | | |
| σ^2^ | 13.32 | | |
| τ_00_ _ResponseId_ | 215.61 | | |
| ICC | 0.94 | | |
| N _ResponseId_ | 198 | | |
| Observations | 395 | | |
| Marginal R^2^ / Conditional R^2^ | 0.013 / 0.943 | | |

*Note*. WBISM = Weight Bias Internalization. CDboth = Group comprising those randomized to the Self-Directed Cognitive Dissonance and Other-Directed Cognitive Dissonance conditions. CDself = Self-Directed Cognitive Dissonance. CDother = Other-Directed Cognitive Dissonance.

**Table S3.**

*Results of Multilevel Model Testing Effect of Time by Condition Interaction on AFAT.*

| *Predictors* | *Estimates* | *CI* | *p* |
| --- | --- | --- | --- |
| (Intercept) | 69.26 | 67.27 – 71.25 | **<0.001** |
| time | -1.05 | -1.48 – -0.63 | **<0.001** |
| control_VS_CDboth | 3.75 | -0.51 – 8.01 | 0.084 |
| CDself_VS_CDother | -3.13 | -7.97 – 1.71 | 0.205 |
| **time * control_VS_CDboth** | 0.94 | 0.03 – 1.84 | **0.042** |
| time * CDself_VS_CDother | -0.88 | -1.90 – 0.15 | 0.093 |
| **Random Effects** | | | |
| σ^2^ | 18.23 | | |
| τ_00_ _ResponseId_ | 194.04 | | |
| ICC | 0.91 | | |
| N _ResponseId_ | 198 | | |
| Observations | 396 | | |
| Marginal R^2^ / Conditional R^2^ | 0.028 / 0.917 | | |

*Note*. AFAT = Anti-Fat Attitudes Scale. CDboth = Group comprising those randomized to the Self-Directed Cognitive Dissonance and Other-Directed Cognitive Dissonance conditions. CDself = Self-Directed Cognitive Dissonance. CDother = Other-Directed Cognitive Dissonance.

**Table S4.**

*Results of Multilevel Model Testing Effect of Time by Condition Interaction on IAT.*

| *Predictors* | *Estimates* | *CI* | *p* |
| --- | --- | --- | --- |
| (Intercept) | -0.37 | -0.42 – -0.33 | **<0.001** |
| **time** | 0.06 | 0.03 – 0.09 | **<0.001** |
| control_VS_CDboth | 0.02 | -0.07 – 0.12 | 0.64 |
| CDself_VS_CDother | -0.1 | -0.21 – 0.01 | 0.063 |
| time * control_VS_CDboth | -0.01 | -0.07 – 0.05 | 0.779 |
| time * CDself_VS_CDother | -0.01 | -0.08 – 0.06 | 0.728 |
| **Random Effects** | | | |
| σ^2^ | 0.09 | | |
| τ_00_ _ResponseId_ | 0.06 | | |
| ICC | 0.39 | | |
| N _ResponseId_ | 197 | | |
| Observations | 387 | | |
| Marginal R^2^ / Conditional R^2^ | 0.039 / 0.414 | | |

*Note*. IAT = Implicit Attitudes Test. CDboth = Group comprising those randomized to the Self-Directed Cognitive Dissonance and Other-Directed Cognitive Dissonance conditions. CDself = Self-Directed Cognitive Dissonance. CDother = Other-Directed Cognitive Dissonance.

**APPENDIX A: Intervention Content**

**Self-Directed Anti-Fat Bias: Advice to Adolescent Girls**

1. I started middle school this year, and luckily some older girls I know from my neighborhood have been really nice and welcomed me into their friend group. They’re all so cool, and I can’t believe I’m saying this, but I think I’m actually friends with the “popular” girls!! They even invited me to start sitting at their lunch table, which is so exciting, but I’ve noticed that none of them actually eat during lunch. When I asked why, one of the girls told me they skip lunch to avoid gaining weight. I definitely want to fit in, and I guess I don’t want to gain weight either, so I’ve started trying to skip lunch too. The thing is, on days when I don’t eat, I have such a hard time concentrating in my afternoon classes because my stomach is rumbling from hunger! My new friends are all so beautiful, and I really like hanging out with them, but I’m not sure if I can keep skipping lunch every day. What should I do?
2. I guess I’m going through puberty (*cringe*) because my body has been changing a LOT over the past few months. I’m not really sure how to feel about it all, but I guess I don’t hate finally having some curves. My issue is that all of a sudden, it’s like I have to deal with a whole new set of rules. Anytime I have dessert or go for seconds at dinner, my mom asks if I “really need it” or if I’m “sure,” like she’s trying to make me feel guilty! It’s not just her either. Anytime my aunts and grandma are around, I swear they comment on everything I put in my mouth, saying things like I’ll “regret it later when it’s on my hips.” I know my mom and my aunts are super careful about what they eat, and I usually eat pretty healthy too, but they’re making me feel so weird! My doctor said my body changes are totally normal for my age, but I feel like every new pound or curve is somehow my fault. I try to ignore my family, but their comments are really starting to get to me, and I can’t help but feel guilty anytime I eat, even when they aren’t around! I guess I could try to eat better, but what I really want is to just not have to think about it every second or justify every bite I take. I’ve tried talking to my mom and my aunts, but they just roll their eyes and say I’m being an “emotional, hormonal teenager.” How can I get them to see how much their comments are bothering me and ask them to stop? Also, if you have any suggestions for how to tune them out and not feel guilty when I eat, please send!!
3. My best friend got really sick over our last school break, and she ended up losing a bunch of weight because of it. Luckily, she’s all better now, but she’s pretty embarrassed about the whole thing and made me promise not to tell anyone at school about it. The thing is, ever since we got back to school, she’s been getting compliments nonstop about how great her “new body” looks. I was happy for her at first, especially because of everything she just went through, but it’s honestly getting so hard to watch her get all this attention and not want some too. I know this is terrible to even think, but I keep daydreaming about getting sick and losing weight, just so I can get noticed and maybe praised a little bit too! I looked up other ways to lose weight, and I think I might order some diet pills just to help me get started. I’m a little nervous, but lots of people in the reviews say they’re safe and work really well, and I won’t go overboard or anything. Am I making a terrible mistake? Is there something wrong with me that I’m so jealous of my friend, or maybe even something I can do to stop feeling like this?
4. My mom and older sister use a dieting app to track everything they eat. It’s annoying that they’re so obsessed with it, and they say it’s just about accountability and health, but I’m pretty sure it’s because they want to stay thin. Now that I finally have a smartphone, they’ve started hinting that I should download the app too, and I’m actually kind of considering it. On the one hand, I definitely don’t want to get fat, and I guess I’m a little excited at the idea of getting to join their diet club or whatever. On the other side, I really don’t want to get totally obsessed with my diet like they are! Should I try it out, or stay away?
5. I have a lot of problems with my self-esteem, especially about my body. I have the habit of feeling inferior to the other girls because for me all are beautiful except me. I hate to be like this. I hate my body, I hate to hate my body, I wanted to feel good, I wanted not to feel insufficient. Everyone I’ve talked to about this says I should try coming up with positive things to say about myself, but as embarrassing as this is to admit, I literally can’t ever think of anything positive. I know you obviously don’t know me, but do you have any ideas for how I can start trying to come up with positive things to say about myself? If you ever feel like you hate your body, are there things you say to yourself that actually help?

**Weight discrimination: arguing against policies**

1. A successful tech company provides several initiatives aimed to promote wellness and work-life balance among employees, including extramural sports teams, which are a company favorite. Although games happen outside of work hours, they facilitate strong camaraderie among employees and have become central to many working relationships. Every year during the new employee recruitment season, the company directors emphasize the need for applicants to fit with the “company culture” in order to be considered for a position. Given that extramural sports are such a cornerstone of the office culture, the directors and other members of the hiring committee prioritize applicants whose body size suggests that they exercise regularly. Leaked emails from this committee emphasize that “new employees are not to be selected for their appearance or athleticism, however, an unathletic or overweight person would probably feel left out if they did not want to participate in sports, thus likely making them a bad fit for [the company].”
2. When drug companies run clinical trials to test the effectiveness of new drugs and determine the proper dosages, they are not required to include participants with a diverse range of body sizes, and, in some cases, they may actually exclude people whose weight or BMI measure above a specific cutoff. It recently became apparent that a medication commonly prescribed to women is only fully effective among individuals whose weight is a specific value *below* that of the average American woman. This means that the medication is not fully effective for the majority of its intended consumers. More specifically, the drug is significantly less effective for those whose weight falls into the 10-pound range above the effective weight cutoff; for individuals whose bodyweight measures above this range, the drug has been shown to be completely ineffective. Aside from adding this warning to the label, there have been no reported efforts to develop a better medication or establish an effective dose of the existing medication for women with higher bodyweight.
3. A popular fitness chain with gym locations all over the U.S. has strict policies regarding the appearance of their instructors. The company rejects applicants that do not meet the specific bodyweight standards and weighs employed instructors frequently. To maintain their strict appearance standards, a company policy requires the suspension or termination of any employee whose bodyweight increases by more than a narrow percentage of their weight when hired. The company justifies these policies by claiming that a major part of the success of their brand is the “aspirational” physique of their instructors, and overweight instructors may lead people to conclude that the workout is ineffective or otherwise inhibit motivation.
4. Physical therapy is a field which aims to treat people with a wide range of mobility issues. To manage an overwhelming volume of patients at one practice, a policy was established to use the weight and BMI of prospective patients to identify and prioritize those who are expected to benefit most from treatment. Individuals seeking physical therapy services who are categorized as overweight or obese are instructed to lose a certain amount of weight to see if their issues resolve prior to scheduling an appointment. Despite the fact that many individuals seek physical therapy services for injuries or other issues that are not related to their bodyweight, the physical therapy practice argues that patients who “prioritize their own health by maintaining a healthy weight” are the priority of the clinic.
5. Popular social media apps use artificial intelligence (AI) and complex algorithms to determine which posts should be distributed widely and which should be flagged, removed, or otherwise minimized on a given platform. In many cases, larger-bodied individuals have had their posts removed for “indecency,” despite adhering to the platform’s rules and posting comparable content to that of smaller-bodied individuals, which are widely shared. For example, one platform has developed an automated system for flagging posts in which more than a specific percentage of an individual’s uncovered body is displayed. In practice, this means that posts featuring individuals in smaller bodies wearing minimal clothing, such as a swimsuit, are far less likely to be flagged or removed than posts with a larger bodied person wearing the same garment, simply because more of their uncovered body is visible. Because of these policies, smaller-bodied individuals can amass greater followings from platform users and receive more opportunities for brand partnerships than larger-bodied individuals with similar content.
